# Cutaneous Wound Healing: An Update from Physiopathology to Current Therapies

**DOI:** 10.3390/life11070665

**Published:** 2021-07-07

**Authors:** Lucas Fernando Sérgio Gushiken, Fernando Pereira Beserra, Jairo Kenupp Bastos, Christopher John Jackson, Cláudia Helena Pellizzon

**Affiliations:** 1 Institute of Biosciences of Botucatu, São Paulo State University—UNESP, São Paulo 18618-689, Brazil; claudia.pellizzon@unesp.br; 2 School of Pharmaceutical Sciences of Ribeirão Preto, University of São Paulo—USP, São Paulo 18618-689, Brazil; fernando.beserra@unesp.br (F.P.B.); jkbastos@fcfrp.usp.br (J.K.B.); 3 Kolling Institute of Medical Research, The University of Sydney at Royal North Shore Hospital, St. Leonard, Sydney 2065, Australia; chris.jackson@sydney.edu.au

**Keywords:** skin, wound healing, wound therapies, topical formulation, dressings, skin substitutes

## Abstract

The skin is the biggest organ of human body which acts as a protective barrier against deleterious agents. When this barrier is damaged, the organism promotes the healing process with several molecular and cellular mechanisms, in order to restore the physiological structure of the skin. The physiological control of wound healing depends on the correct balance among its different mechanisms. Any disruption in the balance of these mechanisms can lead to problems and delay in wound healing. The impairment of wound healing is linked to underlying factors as well as aging, nutrition, hypoxia, stress, infections, drugs, genetics, and chronic diseases. Over the years, numerous studies have been conducted to discover the correct approach and best therapies for wound healing, including surgical procedures and non-surgical treatments such as topical formulations, dressings, or skin substitutes. Thus, this general approach is necessary to facilitate the direction of further studies. This work provides updated concepts of physiological mechanisms, the factors that can interfere, and updated treatments used in skin wound healing.

## 1. Introduction

The skin is the largest organ of humans that acts as the first mechanical barrier between the organism and the external environmental, to protect organism against deleterious agents, control thermal regulation, and regulate water and electrolytes homeostasis [1]. The morphologic structure of skin comprises two layers, the epidermis and dermis [2]. The epidermis is the most external layer of the skin and is divided into four or five sub-layers, depending on the region of the body [2]. Among the cells that constitute the epidermis are by far the major cell type, the keratinocytes, as well as melanocytes, Langerhans cells, and Merkel cells. The dermis is the layer of connective tissue that supports the epidermis, constituted by extracellular matrix proteins (collagens, elastin, proteoglycans, and glycosaminoglycans) synthesized by fibroblasts [3]. If there is a disruption of one or both layers of the skin, the organism starts the wound healing process to regenerate the injured area, involving cellular, molecular and biochemical mechanisms divided in three healing phases: inflammatory, proliferative, and remodeling [4,5]. In this review, we discuss the physiologic mechanism of wound healing, the risk factors that affect healing and provide an update on the main therapies to treat skin wounds.

## 2. Physiology of Skin Wound Healing

**Figure 1:**
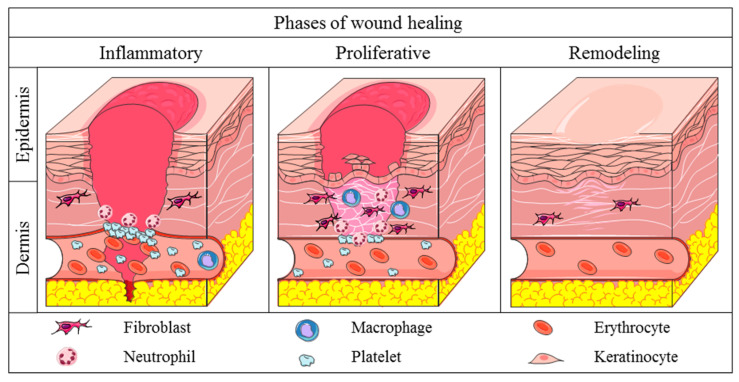
Phases of physiological wound healing. Inflammatory phase: there is the hemostasis of wounded area and acute inflammation through the release of cytokines, growth factors and the migration of leukocytes in the area. Proliferative phase: increase in the migration and proliferation of the keratinocytes, fibroblasts, endothelial cells and leukocytes in the wound. Increase in the synthesis of extracellular matrix components and improve of angiogenesis and re-epithelialization mechanisms. Remodeling phase: extracellular matrix remodeling, with substitution of collagen III for collagen I. Increase in the activity of MMPs. Apoptosis of provisional endothelial cells, fibroblasts, and myofibroblasts of the injury.

Skin wound healing is a complex process involving interrelated and overlapping mechanisms of cell migration and proliferation, synthesis of extracellular matrix, growth factors and cytokines that coordinate the healing process. Due to this complexity, the wound healing process can be divided in three phases: inflammatory, proliferative, and remodeling phases (Figure 1) [6].

The inflammatory phase of wound healing begins with the activation of platelets, which synthesize the compounds responsible for fibrin clot formation, restoring the local hemostasis and acting as a provisional extracellular matrix for the migration of blood cells [5]. Simultaneously, the platelets and injured cells release cytokines and growth factors such as IL-1β (interleukin-1β), TNF-α (tumor necrosis factor-α), FGF (fibroblast growth factor), and PDGF (platelet derived growth factor) which attract leukocytes to the region [4,6]. Initially, neutrophils migrate to the injured area, starting the debridement of necrotic tissue and the phagocytosis of pathogenic antigens. Furthermore, the neutrophils release pro-inflammatory cytokines as IL-1β, TNF-α, IL-6 (interleukin-6) and IL-8 (interleukin-8), which attract other inflammatory cells to the wounded area (Table 1) [7]. The neutrophils also release VEGF (vascular endothelial growth factor) and IGF-1 (insulin growth factor-1) which activate the local proliferation of fibroblasts, keratinocytes, and endothelial cells [7,8]. After a few days, macrophages start to migrate to the wound region to continue the debridement of necrotic tissue and phagocytosis of deleterious antigens, and secrete growth factors and cytokines which coordinate the subsequent mechanisms of the wound healing [5].

The proliferative phase of wound healing is characterized by the intense migration and proliferation of cells and synthesis of granulation tissue, comprised of provisional extracellular matrix, macrophages, endothelial cells, and fibroblasts [10]. The cells in the injury secrete FGF, VEGF, EGF (epidermal growth factor), and TGF-β1 (transforming growth factor-β1) to promote the proliferation of fibroblasts, keratinocytes, and endothelial cells (Table 2). Fibroblasts also synthesize compounds of provisional extracellular matrix, including type III collagen, proteoglycans and fibronectin, in order to support cell migration into the area [5,6]. The restructuring of vascularization at the wound begins immediately after the injury, but has higher activity in the proliferative phase, providing oxygen and nutrients needed for the migration and proliferation of cells and synthesis of extracellular matrix compounds. Secreted mediators as VEGF and angiopoietins stimulate the proliferation of endothelial cells and restructuring of the vascular system at the wound site [11]. During the proliferative phase, re-epithelialization occurs to close the epithelial gap and restores the barrier function of the skin [5,12]. Firstly, the keratinocytes at the border of wounds are stimulated by growth factors, resulting in the proliferation and differentiation of the keratinocytes. This stimulus triggers the loss of keratinocyte adhesion molecules, inhibiting the physical contact with desmosomes and hemidesmosomes, and increasing the migration of these cells through the extracellular matrix [8,12].

The last stage of skin wound healing is the remodeling phase, which depends on the mechanisms started in early phases. There is a decrease of granulation tissue, substitution of provisional extracellular matrix and apoptosis of provisional cells that migrated to the area [5]. The fibroblasts are stimulated by TGF-β1 to differentiate into myofibroblasts, acquiring a contractible phenotype and decreasing the wounded area due to the multiple points of connection of myofibroblast proteins to collagen fibers [14]. Additionally, the proteins of provisional extracellular matrix are degraded by MMPs, metal-dependent proteases synthesized by local cells to remodel the wounded extracellular matrix proteins [15]. Thus, the fibroblasts in remodeling tissue synthesize type I collagen, elastin, and other compounds of permanent extracellular matrix, resulting in higher resistance and flexibility in the regenerated skin [8].

## 3. Pathological Healing 

Wound healing is a physiological process of vertebrates to maintain organism homeostasis and involves perfect interactions of numerous cell types and molecules [5]. The imbalance of these interactions can result in the occurrence of errors in the healing mechanisms and lead to the impairment of wound healing [16].

One of the errors that delays the wound healing is the chronic inflammatory state [17]. Due to the imbalance of pro- and anti-inflammatory mediators, there is an exacerbated recruitment of neutrophils and macrophages to the region, with the overexpression of inflammatory cytokines [18,19]. As result, there is the increase in leukocyte recruitment to the region and the release of reactive oxygen species (ROS) by inflammatory cells [17,20]. The overproduction of ROS inflicts cellular damage, interfering with proliferation/differentiation of keratinocytes and fibroblasts in the wounded area and leading to cell apoptosis [21]. Furthermore, the ROS cause the degradation of growth factors involved in healing mechanism, decreasing the quantity and bioavailability of these molecules [20,21,22]. Moreover, the increased in pro-inflammatory cytokines affects the subsequent mechanisms of wound healing, increasing MMPs and other proteases that impairs cell proliferation/migration and decreases the accumulation of extracellular matrix components [18,23,24].

In physiologic cicatrization, there is the perfect balance between the proliferation/activation and maturation/apoptosis of blood vessel during the proliferative phase of healing [25]. However, the imbalance between pro- and anti-angiogenic factors promotes the decrease of neovascularization and blood flow in the area, delaying the subsequent mechanisms from proliferative and remodeling phases [26].

Delay in the re-epithelialization is another error that can occur in wound healing. As a consequence of chronic inflammation and reduced vascularization, keratinocytes from pathological wound edges acquire a hyperproliferative state because of the overexpression of β-catenin/c-myc pathway [27,28]. Moreover, these keratinocytes differ from normal wound keratinocytes because they are hyperkeratotic and parakeratotic and express low levels of keratins 1, 2 and 10 [29,30,31]. In addition, the migratory potential of the keratinocytes in abnormal healing is impaired [29,31]. The molecular mechanism of this poor migratory potential of keratinocytes is related to the proteolytic degradation of growth factors and extracellular matrix proteins mandatory for the migration, as well as the reduced expression of laminin 3A32, a precursor of laminin 5 associated with keratinocyte migration [12,31].

Impairment in remodeling is other important failure of wound healing. The cells of the injury synthesize excessive amounts of MMPs and other proteases, degrading not only extracellular matrix components, but also cell surface receptors, growth factors, and cytokines [32,33]. In addition, inhibitors of metalloproteinases (TIMPs) are reduced, contributing to protease deregulation in these injuries [32,33]. As a consequence, there is a degradation of important molecules from extracellular matrix such as collagen, elastin, fibronectin and chondroitin sulfate, impairing proliferation and migration of the cells [20,23]. Furthermore, the overproduction of MMPs and the degradation of cytokines and growth factors lead to a cycle of extracellular matrix degradation/synthesis, resulting in the impairment of extracellular matrix remodeling mechanism [20,33].

## 4. Factors That Affect the Wound Healing

**Figure 2:**
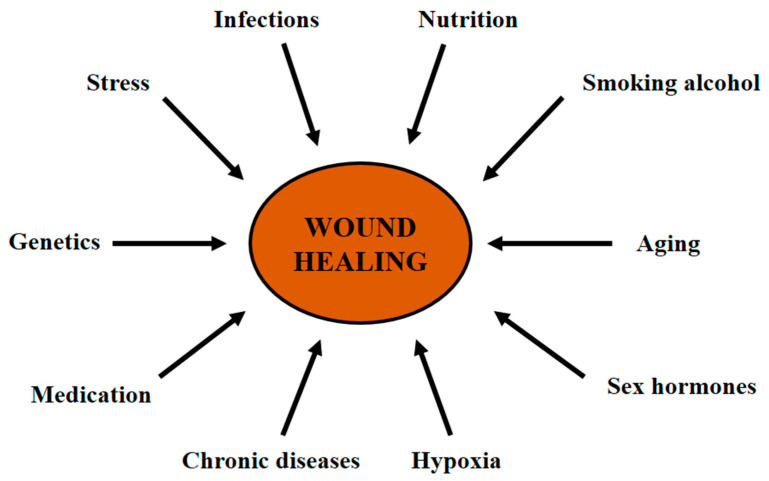
Factors that affect wound healing. Common situations that delay skin wound healing.

The human body is susceptible to numerous local and systemic conditions which can negatively affect skin repair through various mechanisms, leading to a delay in the process. The major conditions that interfere with wound healing are discussed below (Figure 2) [34].

### 4.1. Hypoxia

Oxygen is required for ATP synthesis which is essential for cell metabolism and survival. When an injury occurs, there is a decrease in local oxygen supply due to the vascular disruption [34]. The hypoxic wound microenvironment is important because it provides the release of mediators that coordinate the mechanisms of angiogenesis, re-epithelialization, and synthesis of growth factors and cytokines [34,35,36]. However, hypoxia leads to the synthesis of reactive oxygen species (ROS) and pro-inflammatory cytokines that can impair the healing process. Furthermore, the correct oxygen concentration is necessary to prevent wound infection, improve the angiogenic response, and increase fibroblast and keratinocyte differentiation, proliferation and migration [35]. Therefore, the correct balance of oxygenation is necessary to avoid the impairment and consequent delay of wound healing.

### 4.2. Nutrition

Wound healing requires numerous vitamins, minerals, fatty acids, carbohydrates, and proteins to perform the correct regenerative process [37]. Malnutrition impairs healing by prolonging inflammation, decreasing angiogenesis, phagocytosis and fibroblast metabolism, and prolonging the extracellular matrix remodeling [38]. Some of the essential nutrients that are important for wound healing are the omega-3 fatty acids (modulating the arachidonic acid pathway and cell membrane synthesis), vitamin A (improving the proliferation of keratinocytes), vitamin C, and carbohydrates (responsible for collagen synthesis) [37,38]. Proteins and aminoacids such as arginine, cysteine, methionine, and glutamine modulate the immune cell activity and control the collagen synthesis. Zinc is a cofactor for RNA and DNA biosynthesis, with pivotal role of proliferating cells of wound [39]. Iron act as cofactor in collagen synthesis and its deficiency impairs extracellular matrix remodeling. In addition, iron has important role in oxygen transport and hypoxia, as part of the hemoglobin molecule [37,39].

### 4.3. Infection

When the skin is injured, there is the possibility of local bacterial infection, resulting in the delay of healing process [38]. *Staphylococcus aureus*, *Pseudomonas aeruginosa*, and other *Streptococci* species are largely responsible for wound infection. In response to infection, the human organism starts an inflammatory mechanism, with the migration of leukocytes and cytokine release [34]. However, the phagocytic activity of leukocytes leads to the release of endotoxins by the bacteria, resulting in local necrosis and inflammation due to the increase of pro-inflammatory cytokines, higher metalloproteinase activity and decrease of growth factors release [10,40]. Although an immediate inflammatory response is an initial physiologic mechanism of healing, chronic inflammation impairs the healing process, affecting re-epithelialization and delaying wound retraction and tissue remodeling [10,34].

### 4.4. Stress

Stress can interfere negatively on wound healing, as well as in many systemic diseases, through the deregulation of endocrine hormones. Stress acts in the nervous system and hypothalamus increasing the release of epinephrine, norepinephrine, cortisol and glucocorticoid hormones [34,41]. These molecules promote a decrease of cytokine release and leukocyte immune response, resulting in the impairment of the inflammatory mechanism and the delay of the healing process [41].

### 4.5. Age

Aging is one of the major risk factors related to the impairment of skin wound healing. Due to metabolic and systemic changes during aging, the epidermal layer becomes thinner as people age [37]. There are several alterations in the inflammatory response of elderly people, such as a delay of leukocyte migration to the area, decrease of macrophage phagocytic activity, and reduction of growth factor/cytokine release [37,38]. Furthermore, aged people also present with delayed re-epithelialization, delayed angiogenesis, and a decrease of fibroblast activity and collagen remodeling [34].

### 4.6. Sex Hormones

Estrogen shows anti-inflammatory activity through a decrease of leukocyte infiltration and pro-inflammatory cytokine release [42]. Moreover, estrogen molecules demonstrate influence over keratinocyte and endothelial cell proliferation and migration, increasing the rate of re-epithelialization and angiogenesis of wound healing [43]. In contrast, androgen hormones (testosterone and 5α-dihydrotestosterone) have a chronic inflammatory effect in skin wounds, delaying the healing process by increasing inflammatory cytokines level and leukocyte migration [42,43].

### 4.7. Chronic Diseases

There are many diseases affecting cardiovascular, respiratory, or immunologic systems that can impair skin wound healing by interfering with inflammation, angiogenesis, re-epithelialization, and matrix remodeling mechanisms [38]. Diabetes mellitus, for example, is a multifactorial systemic disease which shows notably impairment of wound healing. The disease affects leukocyte migration and activation and increases pro-inflammatory cytokines release, resulting in chronic inflammation [37]. Diabetes also adversely influences skin microvasculature, leading to a hypoxic environment and decreased angiogenesis. Moreover, the disease modifies keratinocyte and fibroblast proliferation and differentiation, delaying re-epithelialization and extracellular matrix remodeling [37]. Obesity is another chronic disease that causes several complications in skin wound healing. Pressure and venous ulcers associated with hematoma, oedema, seroma formation and local infection are common hallmarks in obese wounds [44,45]. The cellular and molecular mechanisms associated with the impairment of obese wound healing are related to reduced microperfusion at the skin, an excessive release of pro-inflammatory cytokines, and decreased immune response [34,44].

### 4.8. Medication

Currently, there are approved drugs that can have unfavorable influence on wound healing, by interfering with coagulation cascade, inflammatory mechanism, or cell proliferation [34]. Corticosteroids are used routinely as anti-inflammatory agents and also to modulate immune response, but the systemic anti-inflammatory effect of steroids induces a decrease in growth factors and cytokines which modulates other mechanisms of healing, as well as the decreasing of fibroblast proliferation [46]. Non-steroidal anti-inflammatory drugs (NSAIDs) are used systemically to treat inflammation and pain but demonstrate a negative impact on wound healing by decreasing fibroblast proliferation, reducing wound retraction and delaying angiogenesis. When administered topically, NSAIDs formulations improve wound healing and reduce local pain [4,47]. Chemotherapeutic drugs can also interfere with skin wound healing because the mechanism of action of these molecules decreases cellular metabolism and proliferation. As consequence, there is a decrease in re-epithelialization, angiogenesis, collagen synthesis, and delay of wound retraction [46].

### 4.9. Smoking

It is well-known that smoking is related to the increase of risk of several diseases, including the impairment of skin wound healing, with studies proving that cigarette compounds like tobacco, nicotine, carbon monoxide, and hydrogen cyanide affect the mechanisms of healing [48]. Hypoxia is one of the major mechanisms for smoking-related impairment of wound healing, decreasing the proliferation of erythrocytes, oxygenation, blood flow, and angiogenesis in wounded tissue [34]. Smoking also increases platelet aggregation and adhesiveness, increasing blood viscosity which results in a higher risk of thrombosis and embolism. Furthermore, cigarette compounds are involved in the decrease of fibroblast migration, proliferation and collagen remodeling [34,45]. Smoking has influence in immune system, decreasing neutrophil, macrophage, and lymphocyte activity, resulting in a higher risk of infections [34,48].

### 4.10. Alcohol

Chronic or acute intake of alcohol contribute to the impairment in skin wound healing [34]. One of the mechanisms responsible is the suppression of host immunity and increase in susceptibility to infections. Studies revealed the influence of alcohol on inflammation, initially by decreasing neutrophil recruitment/activity and pro-inflammatory cytokines, then subsequently by promoting the chronic elevation of cytokines and leukocyte in the later stages of healing [45]. Moreover, alcohol consumption has an influence on the proliferative phase, reducing angiogenesis in the wounded area through the low expression of VEGF receptors [34,45]. As consequence, a hypoxic environment occurs in the region with the formation of oxidative stress molecules and free radicals. Alcohol intake also impairs the remodeling mechanism, decreasing collagen synthesis and altering the concentration of extracellular matrix metalloproteinases [34].

### 4.11. Genetic Predisposition 

Cutaneous wound healing can be affected by genetic factors that impair the tissue repair. Keloid, for example, is a wound repair condition that has a strong genetic influence, with an increased occurrence in African, Asian, and Hispanic ancestry and minor occurrence in Caucasian population [49]. Moreover, studies suggest that there is a major risk of keloid in mutated genes that overexpress collagen deposition and in susceptible loci, like rs8032158 SNP in *NEDD4* gene on chromosome 15 [34]. Ehlers-Danlos syndrome comprises several disorders with defective connective tissue and collagen synthesis, characterized by skin fragility and hyperflexibility, joint hypermobility, and impaired wound healing [50]. Mutations in *COL3A1* and *COL5A1* genes lead to nonfunctional collagen III and V, with alterations in extracellular matrix remodeling [37,50]. Epidermolysis bullosa is a group of genetic skin diseases characterized by fragility in dermo-epidermal junction and separation of cutaneous layers, resulting in blisters with impaired healing [51]. The existing genetic alterations in all types of epidermolysis bullosa can be found in *COL16A1* (type XVI collagen) and *FN1* (fibronectin) genes, extracellular matrix molecules related to the remodeling mechanism of skin wound healing [37].

## 5. Wound Treatments

According to the World Health Organization, millions of people who suffer with pathologic wounds annually require medical care [52]. Currently, there are several treatments for skin injuries, comprising surgical procedures, non-surgical therapies, and pharmacologic agents, with costs of $12 billion annually and that will reach $35 billion in 2023 [52]. However, depending on the size and type of wound, the existing wound therapies are not effective. Below, we discuss the current and new alternatives to the enhancement of skin wound healing [53].

### 5.1. Surgical Procedures

There are some surgical options to treat wounds, depending on the etiology and type of injury. Skin grafts are the most common surgical alternative to the partial treatment of burns or chronic wounds and can be divided in three different categories: autografts, allografts, and xenografts [54]. The surgical gold standard to the restoration of a wound is the autograft, in which a healthy suitable area of skin from the same person is transplanted to the injured area [55]. The autologous tissue allows the reorganization of local vasculature and restoration of epidermal function and has the advantage of no immune rejection. However, the use of autografts has some limitations, such as insufficient normal skin sites for autologous transplant in wounds that cover a large area, consequent scarring, and painful healing [54,55]. Allografts are an alternative to autograft and can be defined as the transplantation of a suitable skin from another person. On the other hand, xenografts are skin samples from a different animal species that are transplanted to humans [55]. These therapies have many deficiencies involving not only the possibility of scarring and painful healing, but also the possibility of cross-infection and immune rejection of the transplanted tissue [54].

The surgical debridement of devitalized tissue is an alternative to extensive necrotic life-threatening wounds. Debridement of nonviable tissue is important to prepare the wound bed, avoid the impairment of healing and accelerate the extracellular matrix remodeling [56]. This is a fast and effective procedure undertaken with the patient under anesthesia. Although the effectiveness and agility of surgical debridement, this method has increased risks because of the general anesthesia [56].

The use of flaps is other surgical therapy to skin wounds. A skin flap is composed by skin and subcutaneous tissue partly detached and moved to cover a nearby wound, preserving the blood supply [57]. Blood flow maintenance is the main advantage of skin flaps compared with grafts, providing nutrients to cell proliferation/differentiation, cytokines and growth factors that control the healing. However, any microvasculature disturbance may results in flap failure and impairs the healing [58]. The anesthetic-dependence as an invasive therapy is other disadvantage of flaps compared with non-surgical treatments. Then, a high knowledge of skin flaps physiology and biomechanics is essential to the success of this method [58,59].

### 5.2. Non-Surgical Therapies

Current non-surgical therapies for skin wounds include topical formulations, dressings, scaffolds, and skin substitutes. These treatments generally fit into the following categories: manage infection and inflammation; correct balance of the wound moisture and exudate; debride the wound site or control the progress of re-epithelialization and wound contraction [60].

#### 5.2.1. Topical Formulations

Topical dermatological treatments can be defined as the application of a formulated drug to the skin [61]. The topical route of administration is the most commonly used one to treat skin wounds, with several advantages compared to systemic drugs, such as avoidance of first pass metabolism and systemic side effects, easy drug application, and suitability for self-medication. However, depending on the formulation, there is the possibility of discomfort, skin irritation, and allergenic reactions [61,62]. Topical formulations include creams, gels, emulsions, ointments, pastes, suspensions, lotions, foams, and sprays. There are also emerging approaches to improve the stability and efficacy of topical treatments, like particulate carriers, liposomes, nanoparticles, and biopolymers [63,64].

Topical antibiotics have been widely used in skin wounds. Creams and ointments containing neomycin, bacitracin zinc, polymyxin B sulfate, povidone-iodine, metronidazole, or silver sufadiazine act in the prevention and treatment of infections in wounds, with effectiveness against several bacteria such as *Staphylococcus aureus*, *Streptococcus pyogenes*, *Pseudomonas aeruginosa*, *Escherichia coli*, and *Klebsiella pneumoniae* [2,65,66]. The topical antimicrobials were developed to prevent wound infection and its complications by stopping the functions of or destroying the microorganisms [10]. The local route was preferred instead of systemic antimicrobials because of the higher risk of bacterial resistance. Moreover, the use of formulations containing these antibiotics in skin wounds can accelerate the healing process, avoiding water loss through the injury and maintaining the moisture in the microenvironment [65]. These drugs are most effective in the inflammatory phase of wound healing and must be suspended when the wound is free of infection, due to the occurrence of skin hypersensitivity reactions and allergic contact dermatitis. [10,67].

Dexpanthenol, used as emulsions or ointment, is an analogue of pantothenic acid that improves the healing process by controlling the proliferation of fibroblasts and the synthesis of granulation tissue. The molecule also accelerates re-epithelialization, by promoting the proliferation and migration of the keratinocytes from the border of the wounds. Re-epithelialization is dependent on a perfect balance among cytokines, growth factors, and other mediators stimulated by dexpanthenol [68,69]. For this reason, dexpanthenol improves epithelialization, by controlling the proliferative and migratory profile of the keratinocytes from the border of the wounds, with major effectiveness during the proliferative phase of wound healing [69].

Collagenase ointment is the most used enzymatic debriding agent for skin wounds and have been used effectively to treat skin wounds for decades [70]. Collagenase is an enzyme derived from bacteria *Clostridium histolyticum* that breaks down denatured collagen, promoting enzymatic debridement of necrotic tissue and extracellular matrix proteins in full-thickness wounds [60]. Therefore, the collagenase formulation has its best activity with the tissue debridement and extracellular matrix remodeling, during the remodeling phase of wound healing [10]. Besides the debridement mechanism, studies indicate that collagenase ointments increase the proliferation and migration of endothelial cells and keratinocytes, with possible involvement in angiogenesis and re-epithelialization mechanisms [60].

In the last decades, growth factors have been tested topically in wounds because of their benefits in healing. However, conventional formulations have low bioavailability caused by rapid clearance of growth factors [71]. As a result, emerging approaches such as nanoparticles and carriers are combined with topical formulations to improve the stability and bioavailability of growth factors as new treatments [71,72,73]. Becaplermin (Smith & Nephew Inc, Fort Worth, TX, USA) is a recombinant human PDGF dispersed in sodium carboxymethylcellulose-based gel which shows clinical benefits for patients with diabetic foot ulcers, stimulating neutrophil, monocyte, and fibroblast chemotaxis and proliferation [73]. In vitro and in vivo studies involving the topical application of KGF conjugated with gold nanoparticles show the promising healing potential of this formulation, enhancing the re-epithelialization through keratinocytes migration and proliferation [71,72]. Topical FGF also has being used experimentally as skin wound therapy. According to a study carried out by Xu et al. (2020), the topical application of FGF10-loaded microspheres emulsion improves collagen synthesis and angiogenesis [74].

#### 5.2.2. Dressings

Dressings protect the injured area from exogenous agents and mechanical trauma, reducing the risk of infections and accelerating the healing process [10]. In the past, dressings functioned to keep the wound dry, removing the wound exudate. However, nowadays, it has been shown that a warm and moist microenvironment can accelerate healing [75]. Therefore, a good dressing must provide protection to the wounded area, remove the excess of exudate in the wound, allow the gaseous exchange, keep a moist microenvironment in the wound bed, and be easy to use [76]. For this reason, recent dressings were developed in order to overcome the disadvantages of previous. Recent dressings can be classified according to the polymeric materials which they are synthesized, including alginate, films, foams, hydrocolloids, hydrofibers, and hydrogels [62,75,77]. Studies suggest that the polymeric structure of dressing components have positive effects in healing, such as fibroblast proliferation and collagen deposition mediated by calcium alginate dressing, and the antibacterial film resulting from hydrogels and hydrocolloids wound interactions [62,75,78,79].

There is a different type of dressing synthesized with biomolecules that combine tissue engineering and nanotechnologies using biological polymers (collagen, elastin and hyaluronic acid), growth factors and drugs [10,75,80]. These biological dressings mimic extracellular matrix and key molecules in cellular proliferation/migration/differentiation [75]. Natural compounds such as propolis, honey, quercetin, and silk fibroin can be associated with films or nanofibers dressings to improve their biocompatibility, antibacterial potential, remove the excess of exudate, and maintain wound moisture. In addition, in vitro and in vivo experiments confirm the influence of these biological dressings in re-epithelialization and collagen deposition [81,82,83,84]. Antibiotic drugs and composites can be associated with biological dressings to improve their healing activities. Studies involving doxycycline and graphene oxide-associated dressings accelerated wound healing, improving re-epithelialization and collagen remodeling, and enhancing the molecules antibacterial activity [85,86]. Nanotechnologies have been used in dressings for growth factor incorporation, ameliorating growth factors bioavailability and stability. According experimental studies, EGF and FGF- incorporated dressings accelerate angiogenesis, extracellular matrix remodeling and re-epithelialization, with great potential in skin wound treatment [87,88,89,90,91,92].

#### 5.2.3. Skin Substitutes

Skin substitutes mimic the normal skin, providing a protective semi-permeable barrier to the wounded area, enhancing the healing and facilitating skin regeneration [55]. Besides improve the wound healing and decrease the risk of local infections, skin substitutes also reduce the mortality and morbidity associated with chronic wounds [55,93]. Skin substitutes can be synthesized by materials distributed in three categories: scaffolds, growth factors and cells. Three-dimensional scaffolds consist act as an extracellular matrix analogue, allowing the adhesion, proliferation, migration and differentiation of the cells present in the wound [93]. Acellular skin substitutes are scaffolds with no cells, providing a template for keratinocytes, fibroblasts, and endothelial cells from the wound. Some examples of polymers used to synthesize the scaffolds are alginate, chitosan, collagen, elastin, fibronectin, glycosaminoglycans, and hyaluronic acid [93,94,95]. Growth factors (EGF, FGF, PDGF, TGF-β, and VEGF) can be found in skin substitutes, enhancing the proliferation and migration of endothelial cells, fibroblasts and keratinocytes of the wound [55,93]. Alloderm™ and Matriderm^®^ are commercially available cell-free dermal substitutes composed of a three-dimensional lyophilized collagen scaffold, recommended in burn or chronic wounds [96]. Integra™ and Biobrane^®^ are also acellular dermal substitutes with a three-dimensional type I collagen and chondroitin sulfate scaffold as extracellular matrix analog and a silicone film as epidermis analog. These commercial skin substitutes are recommended in the treatment of partial and full thickness wound, burns, or chronic ulcers [96,97].

Cellular skin substitutes have cells in the constitution and although the cells are not necessary to synthesize the skin substitutes, the addition of fibroblasts, keratinocytes, macrophage, endothelial cells, and stem cells improve the control of the regenerative mechanisms [98,99,100]. Bioseed-S^®^, Epicel^®^, EpiDex^®^, and MySkin™ are cellular epidermal skin substitutes composed by an autologous cultured keratinocyte layer seeded on scaffolds (silicone, petrolatum gauze, allogeneic fibrin), mimicking the epidermal barrier [97,101]. As epidermis analogues, these therapies are suitable as temporary wound coverage in patients who lose a big part of their total body surface area, accelerating the wound re-epithelialization. However, there are some disadvantages such as long-time preparation, hyperkeratosis and scar possibility [55,97]. When it is necessary a greater mechanical stability, dermal substitutes are more suitable than epidermal [101]. Dermagraft™ and Transcyte™ are cellular commercial dermal substitutes composed by cultured allogeneic neonatal skin fibroblasts seeded in type I collagen/silicone film [55,96,97]. These extracellular matrix analogues and their fibroblasts secrete growth factors and proteins that enhance reepithelialization by the patient’s keratinocytes in partial- and full-thickness wounds [55]. In some injuries, the treatment with epidermal (keratinocyte) or dermal (fibroblast) substitutes alone can result in improper healing. Thus, more sophisticated cellular skin substitutes have been developed, mimicking epidermal and dermal layers and providing growth factors, cytokines and extracellular matrix components for initiation/regulation of wound healing [101]. Apligraf™ and OrCel™ are two commercial products containing a type I collagen scaffold seeded with allogeneic neonatal skin keratinocytes and fibroblasts [102,103]. Both therapies are used in partial and full thickness burns, chronic wounds and diabetic foot ulcers, stimulating wound repair through FGF- and KGF-related mechanisms [55,97]. Alternatively, Tiscover™ (A-Skin) consists of an autologous full-thickness cultured skin, used in the treatment of chronic therapy-resistant wounds [55,97]. The major disadvantages of dermo-epidermal cellular substitutes are their high cost and the possibility of tissue rejection, with necessity of new studies and technologies to overlap these problems [101].

## 6. Conclusions

The use of different therapies to treat skin wounds and the development of new drugs for wound healing requires the comprehension of the physiology of normal healing and the alterations in pathological healing. As discussed in this review, skin wound healing is composed of overlapping and interdependent mechanisms—re-epithelialization, inflammation, angiogenesis, wound contraction, and extracellular matrix remodeling—and the imbalance among the factors that control these mechanisms can lead to a delay in the healing process, or even to unhealed wounds. Consequently, the understanding of the mechanisms that comprise the healing process, the factors that can affect the healing and the different options of therapies to wounds can provide a strong basis for the development of new drugs and technologies to skin wound treatment. Nowadays, the most used therapies for skin wounds are topical formulations containing antimicrobial, anti-inflammatory, or debriding drugs and topical growth factors improved by nanoparticles and nanostructured carriers. Furthermore, biological molecules and nanotechnology have been used to synthesize recent dressings and skin substitutes to obtain better results in tissue regeneration. Future perspectives in skin wound healing should include therapies that comprise mechanisms of all healing phases. Numerous advances have been made in the last years in order to discover new drugs, biocompatible dressings and non-immunogenic skin substitutes. With tissue engineering advances, stem cells technologies, bioinformatics and miRNAs regulation, more studies are required for the development of safe, efficient, and low-cost wound therapies.

## Figures and Tables

**Table 1:** Main cytokines involved in the inflammatory phase of skin wound healing. Adapted from Holloway et al., 2011 [9].

Cytokine	Secretory Cells	Biologic Effect
Pro-inflammatory cytokines		
IFN-γ	Macrophage, neutrophil, lymphocyte T	Macrophage activation/Decrease of collagen synthesis/Synthesis of MMPs
IL-1β	Macrophage, neutrophil, keratinocyte	Chemotaxis of fibroblast and keratinocyte/Synthesis of MMPs
IL-6	Macrophage, keratinocyte neutrophil	Chemotaxis of macrophages and neutrophils/Proliferation of fibroblasts
IL-8	Macrophage, neutrophil, fibroblast	Chemotaxis of macrophages and neutrophils/Synthesis of collagen
TNF-α	Macrophage, neutrophil	Cytotoxicity of macrophages and neutrophils/Synthesis of MMPs
Anti-inflammatory cytokines		
IL-4	Lymphocyte T, basophils, mast cells	Decrease of TNF-α, IL-1β and IL-6/Proliferation of fibroblasts/Synthesis of collagen
IL-10	Lymphocyte T, macrophage, keratinocyte	Decrease of TNF-α, IL-1β and IL-6/Inhibition of macrophages and neutrophils

**Table 2:** Growth factors involved in skin wound healing. Adapted from Bennett et al., 2003 [13].

Growth Factor	Secretory Cells	Biological Effect
EGF	Macrophages/Keratinocytes	Proliferation of fibroblasts and keratinocytes
FGF-2	Fibroblasts/Endothelial cells	Proliferation of fibroblasts and keratinocytes
IGF-1	Fibroblasts/Endothelial cells/Neutrophils	Proliferation and differentiation of keratinocytes, fibroblasts and endothelial cells
KGF	Fibroblasts	Proliferation and migration of keratinocytes
PDGF	Macrophages/Platelets	Activation of neutrophils and fibroblasts/Proliferation of fibroblasts and endothelial cells
TGF-β1	Platelets/Macrophages/Fibroblasts/Keratinocytes/Neutrophils/Endothelial cells	Angiogenesis/Extracellular matrix remodeling/Fibroblast differentiation
VEGF	Neutrophils/Endothelial cells/Platelets	Angiogenesis

## Data Availability

No new data were created or analyzed in this study. Data sharing is not applicable to this article.
